# *N*-Acetylcysteine in Mechanically Ventilated Rats with Lipopolysaccharide-Induced Acute Respiratory Distress Syndrome: The Effect of Intravenous Dose on Oxidative Damage and Inflammation

**DOI:** 10.3390/biomedicines9121885

**Published:** 2021-12-12

**Authors:** Maros Kolomaznik, Pavol Mikolka, Juliana Hanusrichterova, Petra Kosutova, Katarina Matasova, Daniela Mokra, Andrea Calkovska

**Affiliations:** 1 Biomedical Centre Martin, Jessenius Faculty of Medicine in Martin, Comenius University in Bratislava, 03601 Martin, Slovakia; maros.kolomaznik@uniba.sk (M.K.); petra.kosutova@uniba.sk (P.K.); 2 Department of Physiology, Jessenius Faculty of Medicine in Martin, Comenius University in Bratislava, 03601 Martin, Slovakia; pavol.mikolka@uniba.sk (P.M.); topercerova4@uniba.sk (J.H.); daniela.mokra@uniba.sk (D.M.); 3 Clinic of Neonatology, Jessenius Faculty of Medicine in Martin, Comenius University in Bratislava and Martin University Hospital, 03601 Martin, Slovakia; matasova.katka@gmail.com

**Keywords:** ARDS, bacterial lipopolysaccharide, *N*-acetylcysteine, lung functions parameters, inflammation, oxidative damage

## Abstract

Treatment of acute respiratory distress syndrome (ARDS) is challenging due to its multifactorial aetiology. The benefit of antioxidant therapy was not consistently demonstrated by previous studies. We evaluated the effect of two different doses of intravenous (i.v.) *N*-acetylcysteine (NAC) on oxidative stress, inflammation and lung functions in the animal model of severe LPS-induced lung injury requiring mechanical ventilation. Adult Wistar rats with LPS (500 μg/kg; 2.2 mL/kg) were treated with i.v. NAC 10 mg/kg (NAC10) or 20 mg/kg (NAC20). Controls received saline. Lung functions, lung oedema, total white blood cell (WBC) count and neutrophils count in blood and bronchoalveolar lavage fluid, and tissue damage in homogenized lung were evaluated. NAC significantly improved ventilatory parameters and oxygenation, reduced lung oedema, WBC migration and alleviated oxidative stress and inflammation. NAC20 in comparison to NAC10 was more effective in reduction of oxidative damage of lipids and proteins, and inflammation almost to the baseline. In conclusion, LPS-instilled and mechanically ventilated rats may be a suitable model of ARDS to test the treatment effects at organ, systemic, cellular and molecular levels. The results together with literary data support the potential of NAC in ARDS.

## 1. Introduction

Acute respiratory distress syndrome (ARDS) is a common cause of respiratory failure in critically ill patients. ARDS is characterized by rapid onset, and severe local followed by systemic inflammation, poor oxygenation, hypoxemia, increased alveolar-vascular permeability, lung oedema and pulmonary infiltrates [1,2]. Incidence of ARDS ranges from 6.3 to 7.2 cases/100,000 population/year and is associated with high morbidity and mortality [3,4,5]. ARDS is multifactorial syndrome and it is often modelled by lipopolysaccharide (LPS) administration in the lungs [6]. LPS, also known as endotoxin, is a part of the outer membrane of Gram-negative bacteria and it has been previously used to induce ARDS in rodents [7,8]. In the respiratory system LPS binds to toll-like receptor (TLR) complex CD14/TLR4/MD-2 located on host cellular membranes and can also play a crucial role in bacteria-host interactions by modulating responses by the host immune system [9]. LPS can induce severe inflammation, oxidative stress and pulmonary oedema by increasing proinflammatory cytokine production, oxidative damage to lipids and proteins and alveolar-capillary barrier permeability [8]. LPS-induced activated alveolar macrophages secret proinflammatory cytokines, such as tumour necrosis factor-α (TNF-α), interleukin-1α (IL-1α), and IL-6. These proinflammatory cytokines increase recruitment and infiltration of inflammatory cells, especially neutrophils, into the damaged lung tissue. Recruited and activated neutrophils also secret various toxic molecules, including oxygen free radicals, which can result in damage of capillary and alveolar endothelia [10]. In addition, LPS affects translocation of the transcription factors p65 NF-κB and signal transducer and activator of transcription 3 (STAT3) from the cytoplasm to the nucleus, triggering the transcription of several proinflammatory genes responsible for cytokine storm [11]. Therapeutic protocol for ARDS patients is based on lung-protective ventilation, fluid-restrictive strategies, and prone positioning, with emphasis on iatrogenic lung injury prevention [12]. Variety of pharmacologic interventions, including glucocorticoids, exogenous surfactants, inhaled nitric oxide, antioxidants, protease inhibitors, other anti-inflammatory drugs etc., have been tested in the ARDS conditions, whereas convincing evidence has been provided for glucocorticoids [1,13,14,15,16]. The multifactorial aetiology of the syndrome is thought to be responsible. Therefore, still open question is to find the suitable therapy for ARDS. *N*-acetylcysteine (NAC) is a thiol compound, precursor of cellular glutathione (GSH) synthesis, which easily penetrates cell membranes and is considered to be “virtually non-toxic” [17]. It can act both as a precursor of reduced glutathione and as a direct reactive oxygen species (ROS) scavenger in the cells. In this way, it can interfere with several signalling pathways that play a role in the regulation of apoptosis and inflammatory response [18]. NAC is capable of restoring the pro-oxidant/antioxidant balance and therefore has been commonly used as the efficient antioxidant [19]. *N*-acetylcysteine is an antioxidant that has been in clinical use primarily to reduce hepatic injury in the settings of paracetamol (acetaminophen) overdose. Currently, intravenous NAC may be considered a potential therapeutic agent in the treatment of severe COVID-19 cases [20]. NAC is transported across the cell membrane where it acts as a source of cysteine for the synthesis of the tripeptide antioxidant glutathione [21].

There are only few animal studies in the literature regarding the use of NAC in ARDS conditions. In addition, the effect of NAC has been evaluated in spontaneously breathing animals. In our study, we evaluated the intravenous effect of two different doses of NAC on oxidative stress, inflammatory tissue damage and lung functions in recently developed model of LPS-induced lung injury requiring mechanical ventilation and oxygen treatment, as this model is supposed to reflect more realistically the situation of the ICU patients.

## 2. Materials and Methods

### 2.1. Animals

In this case, 26 adult male rats (Wistar) with body weights (b.w.) 325 ± 25 g were obtained from VELAZ Animal Breeding Station in Czech Republic. Animals were housed in transparent plastic cages with bedding, five per cage, enhanced by plastic tubes, at a temperature of 20–24 °C and 55 ± 10% and under 12/12 h light/dark cycle. Rats were fed according to the weight range once per day by standard diet (VELAZ) with water ad libitum.

### 2.2. Chemicals

#### 2.2.1. Lipopolysaccharide

Lipopolysaccharide (LPS) purified lyophilized phenol extract of *Escherichia coli* (O55:B5, Santa Cruz Biotechnology, Inc., Dallas, TX, USA) was dissolved in sterile saline. The stock solution of LPS was prepared at concentrations of 500 mg/mL.

#### 2.2.2. *N*-Acetylcysteine

*N*-acetylcysteine (NAC) (ACC Injekt, Salutas Pharma GmbH, Barleben, Germany) was dissolved in sterile saline and given intravenously (i.v.) at a dose of 10 and 20 mg/kg of b.w.

### 2.3. Study Design and Experimental Groups

The animals were instrumented in accordance with previous study [22]. All animals were placed on the heating plate regulated the temperature according to body temperature measured continuously in the rectum (Physitemp Instruments, Inc., TCAT-2LV, Clifton, NJ, USA). After initial anaesthesia with intraperitoneal (i.p.) administration of ketamine (90 mg/kg) and xylazine (10 mg/kg), the animals were tracheotomised and an endotracheal tube was inserted, catheters were placed into the a. femoralis for blood sampling and v. femoralis for continuous anaesthesia infusion (ketamine, 60 mg/kg/h i.v.). Subsequently, the animals were mechanically ventilated (using SLE, SLE5000, Croydon, UK) with tidal volume (V_T_) 6 mL/kg, positive end-expiratory pressure (PEEP) 0.3 kPa, inspiration time (Ti) 40%, fraction of inspired oxygen (FiO_2_) 0.4 and respiratory rate (RR) 60 breaths per minute (bpm). After 15 min of stabilization, LPS at 500 µg/kg b.w. and volume dose of 2.2 mL/kg or saline at the same volume heated to 37 °C were administered intratracheally (i.t.) through a tracheal cannula with a thin short catheter on the syringe cone while positioning the animal to the right and to the left (50% of the dose was given in each position). Lung injury was specified as reduction of dynamic compliance >30% or decrease of P/F (a ratio between arterial partial pressure of oxygen and fraction of inspired oxygen) <40 kPa what indicates mild lung injury. After lung injury, animals were assigned randomly to the following groups: (1) LPS without any treatment (LPS group, *n* = 6); (2) LPS followed by i.v. *N*-acetylcysteine 10 mg/kg b.w. (NAC10 group, *n* = 7); (3) LPS followed by i.v. *N*-acetylcysteine 20 mg/kg b.w. (NAC20 group, *n* = 7) and (4) control animals receiving saline instead of LPS (C group, *n* = 6). All animals were ventilated for additional 4 h. During experiment, gas exchange (PaO_2_, PaCO_2_) and parameters of acid-base balance were measured in arterial blood using blood gas analyzer (Rapidlab TM348 Bayer Diagnostics, Erlangen, Germany).

At the end of the experiment, animals were overdosed by anesthetics. The lungs were excised post-mortem. The left lung lobes were lavaged two times by sterile saline (10 mL/kg b.w.). The recovered bronchoalveolar lavage fluid (BALF) was centrifuged at 1500× *g* for 15 min, supernatant was removed and immediately frozen at −70 °C for further analysis. Tissue samples from right lung were used to assess the degree of lung oedema formation, or used for the biochemical analysis after washing in the cold phosphate buffered saline (PBS, 0.01 M) and homogenized to final concentration 10% (weight/volume) and stored at −70 °C for biochemical analyses.

### 2.4. Evaluation of Lung Functions

P/F was calculated as a ratio between partial pressure of oxygen in arterial blood (PaO_2_) and fraction of inspired oxygen (FiO_2_); ventilation efficiency index (VEI) was calculated due to formula VEI = 3800/[(PIP − PEEP) × frequency × PaCO_2_]; oxygenation index (OI) as (Mean airway pressure × FiO_2_)/PaO_2_ and alveolar–arterial gradient (AaG) as [FiO_2_ (P_atm_ − PH_2_O) − PaCO_2_/0.8] − PaO_2_, where P_atm_ is barometric pressure and PH_2_O is pressure of water vapor.

### 2.5. Evaluation of Lung Oedema

Parts of the wet right lung were weighed before and after being dried at 60 °C for 24 h. Lung oedema was expressed as wet/dry (W/D) lung weight ratio.

### 2.6. Total White Blood Cell Count

Total white blood cell count was evaluated in arterial blood and BALF using veterinary haematology analyser Sysmex XT-2000i (Sysmex, Landskrona, Sweden) and expressed as percentage of basal values.

### 2.7. Assays

To evaluate the oxidative damage the concentrations of malondialdehyde (MDA) and 3-Nitrotyrosine (3NT; both Cell Biolabs Inc., San Diego, CA, USA) were determined in homogenized lung and expressed in µmol/mL (MDA) and nM (3NT). Intercellular adhesion molecule-1 (ICAM1; Abcam, Cambridge, UK), 4-hydroxynonenal (HNE) and myeloperoxidase (MPO; both Cusabio Biotech Co., Wuhan, China) were analysed. Interleukins (IL): IL-1α, IL-1β, IL-4, IL-2, IL-5, IL-6, IL-10, IL-12p70 and IL-13; granulocyte-macrophage colony-stimulating factor (GM-CSF); interferon γ (INFγ); tumour necrosis factor α (TNFα) were evaluated using commercially available kit (Bio-Rad Laboratories, Hercules, CA, USA). All investigated markers were evaluated in homogenized lung at the end of the experiments.

### 2.8. Statistical Analysis

The statistical analysis was performed using GraphPad Prism 6.04 (San Diego, CA, USA). Two-way analysis of variance (ANOVA) with Tukey’s multiple comparison test was used for P/F VEI, OI and AaG. Between-group differences were analysed using Mann-Whitney non-parametric test (LPS vs. C; LPS vs. NAC10; LPS vs. NAC20, LPS vs. NAC10 and NAC10 vs. NAC20). A value of *p* < 0.05 was considered to be statistically significant. All data are shown as mean ± standard deviation (SD).

## 3. Results

### 3.1. Lung Function Parameters

**Figure 1:**
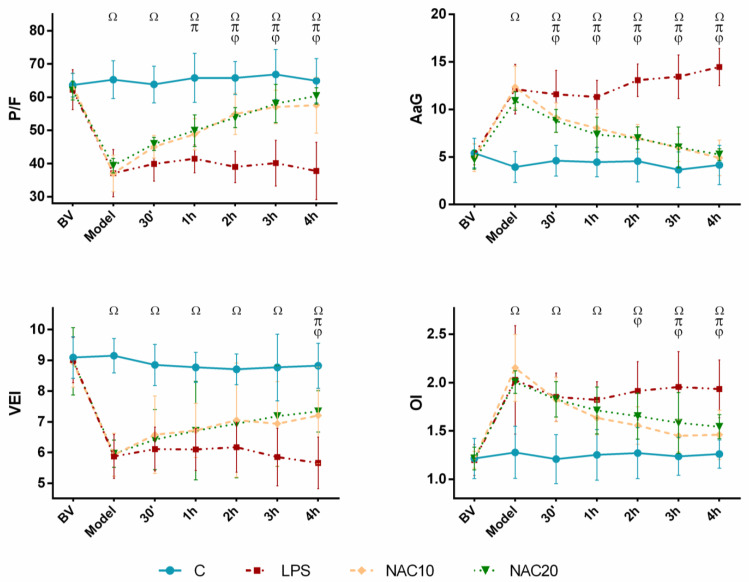
Respiratory parameters: the ratio of arterial oxygen partial pressure to fraction of inspired oxygen (P/F), alveolar-arterial gradient (AaG), ventilation efficiency index (VEI), oxygenation index (OI) before saline/LPS administration (basal value, BV) till experimental model condition (Model) and next 4 h in Control group (C), LPS untreated group (LPS), and LPS groups treated with 10 mg/kg of *N*-acetylcysteine (NAC10) or 20 mg/kg of *N*-acetylcysteine (NAC20). Data are presented as means ± SD. Statistical comparisons: for LPS vs. C ^Ω^ *p* < 0.001; for NAC10 vs. LPS ^φ^ *p* < 0.048 to 0.001; NAC20 vs. LPS ^π^ *p* < 0.032 to 0.001 and for NAC10 vs. NAC20 *p* > 0.05.

Administration of LPS significantly deteriorated lung function expressed by ratio between partial pressure of oxygen in arterial blood to fraction of inspired oxygen (P/F), alveolar–arterial gradient (AaG), ventilation efficiency index (VEI) and oxygenation index (OI) in comparison to control group throughout the whole experiment (for all *p* < 0.001). Both NAC therapies significantly improved P/F ratio, AaG, VEI and OI compared to LPS animals without treatment. The effects between two different doses of NAC (10 and 20 mg/mL) were not significant (Figure 1).

### 3.2. Total White Blood Cell Count in the Arterial Blood

**Figure 2:**
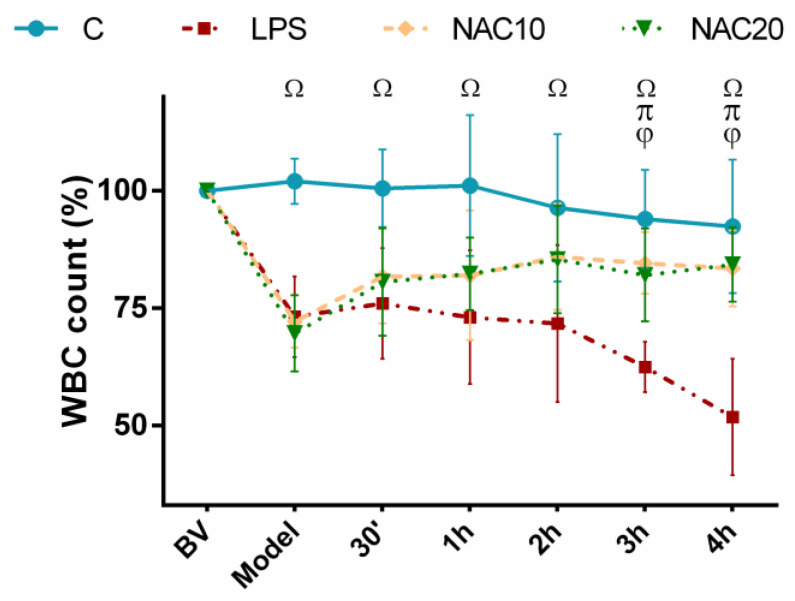
Percentage of total white blood cell (WBC) count in arterial blood before (basal value, BV), after LPS administration (Model) and during 4 h in the course of experiment in Control group, LPS untreated group, and LPS groups treated with 10 mg/kg of *N*-acetylcysteine (NAC10) or 20 mg/kg of *N*-acetylcysteine (NAC20), expressed in percentage (%). Data are presented as means ± SD. Statistical comparisons: for LPS vs. C ^Ω^ *p* < 0.001; for NAC10 vs. LPS ^φ^ *p* < 0.001; NAC20 vs. LPS ^π^ *p* < 0.003 to 0.001 and for NAC10 vs. NAC20 *p* > 0.05.

There were no significant differences in total white blood cell (WBC) count in the arterial blood among the groups at the beginning of the experiment (basal value, BV). Administration of LPS led to significant decrease in the total WBC count in all groups (for all *p* < 0.001 vs. Control). While in the LPS group with no further treatment WBC continuously decreased during whole experiment, WBC count continuously raised after the treatment with both NAC doses and reached almost the levels of control animals (NAC10 vs. LPS *p* = 0.003, NAC20 vs. LPS *p* = 0.001) (Figure 2).

### 3.3. Percentage of Neutrophils in the Arterial Blood and Bronchoalveolar Lavage Fluid (BALF)

**Figure 3:**
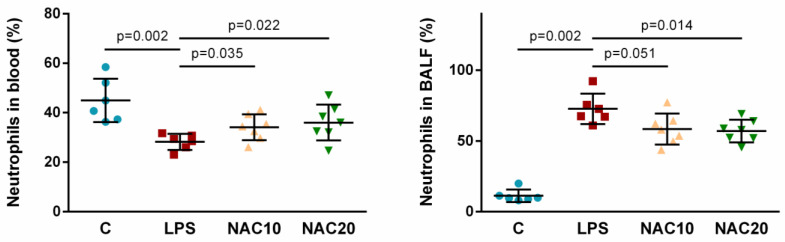
Percentage of neutrophils in the arterial blood and in the BALF at the end of the experiment in Control group, LPS untreated group, and LPS groups treated with 10 mg/kg of *N*-acetylcysteine (NAC10) or 20 mg/kg of *N*-acetylcysteine (NAC20). Data are presented as individual values with means ± SD. Statistical comparisons: for LPS vs. C, NAC10 and NAC20 vs. LPS, NAC10 vs. NAC20.

LPS administration led to the shift of the neutrophils between intravascular and lung compartments. After LPS, percentage of neutrophils in the arterial blood decreased (LPS vs. C *p* = 0.002) and in the BALF increased (LPS vs. C *p* = 0.002). NAC at both doses caused significant elevation of the neutrophils in the blood (NAC10 vs. LPS *p* = 0.035; NAC20 vs. LPS *p* = 0.022) (Figure 3) and in the BALF only NAC20 significantly reduced the percentage of neutrophils compared to LPS animals (NAC10 vs. LPS *p* = 0.051; NAC20 vs. LPS *p* = 0.014) (Figure 3). There was no significant difference in neutrophils distribution between different NAC therapies (NAC10 vs. NAC20 *p* >0.05).

### 3.4. Evaluation of Lung Oedema

**Figure 4:**
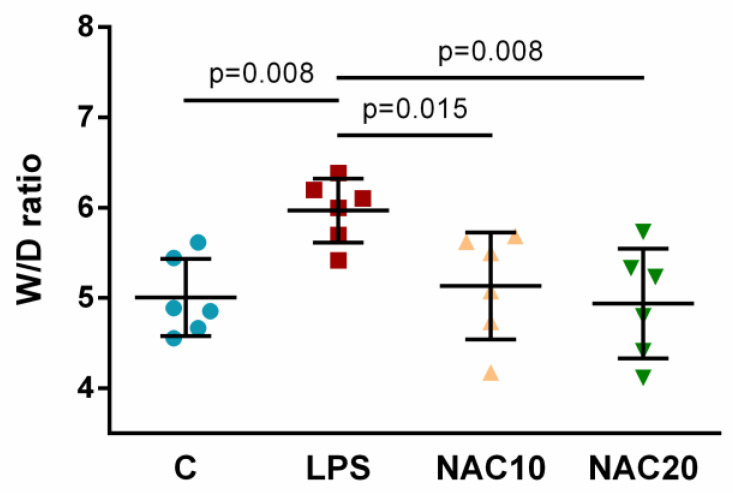
Extent of lung oedema at the end of the experiment. Values represent W/D (wet/dry) weight ratio of lung tissue. Data are presented as individual values with means ± SD. Statistical comparisons: for LPS vs. C, NAC10 and NAC20 vs. LPS, NAC10 vs. NAC20.

Instillation of LPS significantly increased lung oedema formation determined as wet-dry lung weight ratio (W/D ratio) compared to control group (LPS vs. C *p* = 0.008). Administration of NAC significantly reduced lung oedema formation (NAC10 *p* = 0.015; NAC20 *p* = 0.008) vs. LPS group. There were no significant differences between NAC10 and NAC20 (Figure 4).

### 3.5. Oxidative Damage of the Lungs

**Figure 5:**
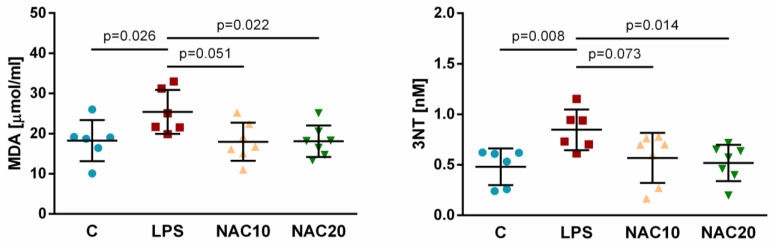
The values of Malondialdehyde (MDA) and 3-Nitrotyrosine (3NT) in lung tissue. Data are presented as individual values with means ± SD. Statistical comparisons: for LPS vs. C, NAC10 and NAC20 vs. LPS, NAC10 vs. NAC20.

Intratracheal LPS increased oxidative damage of lipids in homogenized lung tissue compared to control animal (LPS vs. C *p* = 0.026). Higher dose of NAC significantly reduced the levels of Malondialdehyde (MDA) in comparison with LPS group (NAC20 vs. LPS *p* = 0.022), while administration of low dose NAC oscillated on the border of significance (NAC10 vs. LPS *p* = 0.051). The differences between NAC20 and NAC10 on oxidative lipid damage were nonsignificant (*p* > 0.05; Figure 5).

LPS also increased oxidative damage of proteins in the lungs expressed as levels of 3-Nitrotyrosine (3NT) (LPS vs. C *p* = 0.008). NAC at higher dose, but not at lower dose, significantly reduced the level of 3NT (NAC20 vs. LPS *p* = 0.041 and NAC10 vs. LPS *p* = 0.073). There were no significant differences between both NAC doses (NAC10 vs. NAC20 *p* > 0.05; Figure 5).

### 3.6. Inflammatory Markers

**Figure 6:**
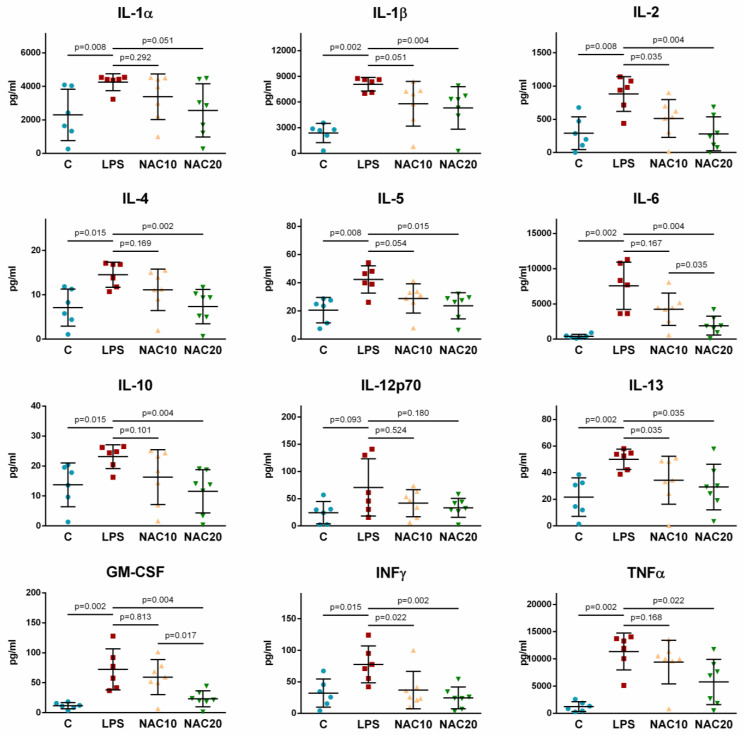
Levels of interleukin (IL)-1α, IL-1β, IL-2, IL-4, IL-5, IL-6, IL-10, IL-12p70, IL-13, granulocyte-macrophage colony-stimulating factor (GM-CSF), interferon γ (INFγ), tumour necrosis factor α (TNFα) in homogenized lung. Data are presented as individual values with means ± SD. Statistical comparisons: for LPS vs. C, NAC10 and NAC20 vs. LPS, NAC10 vs. NAC20.

The levels of all investigated markers significantly increased in LPS group compared to the control group (all *p* < 0.05 to 0.002) except for IL-12p70 (LPS vs. C *p* = 0.093). Compared to LPS group, administration of NAC10 significantly reduced the levels of IL-2, IL-13, INFγ (for all *p* = 0.035 to 0.022). IL-1β (*p* = 0.051) and IL-5 (*p* = 0.054) oscillated on the border of significance. NAC at higher dose 20 mg/mL reduced the levels of IL-1β, IL-2, IL-4, IL-5, IL-6, IL-10, IL-13, GM-CSF, INFγ, TNFα (all *p* = 0.035 to 0.002) with IL-1α (*p* = 0.051) at borderline significance. Differences between NAC groups (NAC10 vs. NAC20) were significant for IL-6 (*p* = 0.035) and GM-CSF (*p* = 0.017) (Figure 6).

### 3.7. ICAM1, MPO, HNE

**Figure 7:**
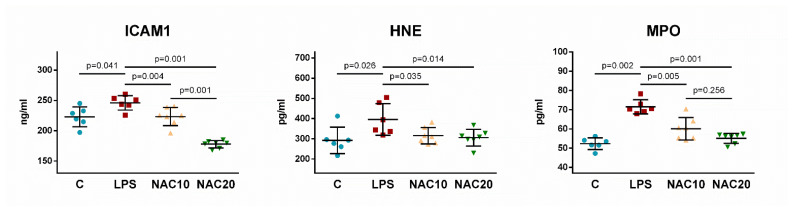
Levels of intercellular adhesion molecule-1 (ICAM1), 4-hydroxynonenal (HNE) and myeloperoxidase (MPO) in homogenized lung at the end of the experiment in Control (C) group, LPS untreated group, and LPS groups treated with 10 mg/kg of *N*-acetylcysteine (NAC10) or 20 mg/kg of *N*-acetylcysteine (NAC20). Data are presented as individual values with means ± SD. Statistical comparisons: for LPS vs. C, NAC10 and NAC20 vs. LPS, NAC10 vs. NAC20.

Intercellular adhesion molecule-1 (ICAM1), 4-hydroxynonenal (HNE) and myeloperoxidase (MPO) significantly raised in non-treated animals with LPS (LPS vs. C, for all *p* = 0.041 to 0.002) in lung tissue. Administration of NAC reduced these markers significantly (vs. LPS all *p* = 0.035 to 0.001) with stronger effect of NAC20 vs. NAC10 for ICAM1 (*p* = 0.001) (Figure 7).

## 4. Discussion

Lipopolysaccharide (LPS) is often used to induce animal models of ARDS LPS binds to TLR4 receptor complex on cell membranes, increases activation of NF-κB and subsequent proinflammatory and prooxidative pathways [23] followed by ARDS-like tissue changes [24,25]. In this study, acute inflammation induced by intratracheal LPS at 500 µg/kg b.w. had negative impact on the lung functions within about 30 min after LPS instillation, as demonstrated by significant drop in ratio of arterial oxygen partial pressure to fraction of inspired oxygen and ventilation efficiency index, and rise in alveolar-arterial gradient and oxygenation index. Intravenous NAC significantly improved the lung functions and this effect was seen also at the end of the experiment. There were no differences between the low (10 mg/kg b.w., NAC10) and high (20 mg/kg b.w., NAC20) dose of NAC in terms of lung functions (Figure 1).

In clinical practice, NAC is used in oral, inhalation and intravenous form. NAC readily crosses the phospholipid bilayer of the cell plasma membrane after administration. Inhaled NAC has low level of internal reducing activity which discourages the wider use of nebulized NAC [26]. Orally administered NAC is rapidly absorbed in the small intestine, reaching peak plasma concentrations 0.5–1 h after administration. Subsequently, NAC is metabolized in the liver to cysteine, which is further used by liver for GSH synthesis. The oral bioavailability of NAC is low (<5%), probably due to metabolic changes in the intestinal wall and hepatic metabolism as well as rapid diffusion into cells and conversion to GSH [27]. In our study, the effect of NAC on lung functions, especially alveolar-arterial gradient, was very fast, seen already 30 min after NAC (Figure 1). With i.v. NAC administration, both first-pass liver metabolism and intestine wall metabolism are bypassed, making it possible to rapidly achieve sufficient NAC concentrations.

Oxidative stress and inflammation are strictly inter-related key processes in the development of lung injury after different insults. LPS acts as a potent chemoattractant for polymorphonuclear neutrophils (PMN) [28] and higher counts of neutrophils may be observed in the lung within several hours after LPS instillation [29]. In this study, the systemic effect of intratracheally administered LPS was demonstrated by reduction of relative numbers of WBC in the peripheral blood continuously throughout the whole experiment (Figure 2). It could be due to neutrophils migration through the capillary wall into the interstitium, and further into the alveoli (Figure 3). NAC caused the shift of the white blood cells, mainly neutrophils, between peripheral blood and the airspaces. Reduced neutrophil count in the alveoli after NAC was seen also in other models of lung injury [30,31]. In contrary, NAC showed no effect on neutrophils in the BAL fluid in model of lung contusion [32], as well as in LPS-induced lung injury [33]. The influx of PMNs into the LPS-challenged lung is associated with production of a wide range of bioactive substances. Activated leukocytes generate also ROS, which may directly injure the lung tissue.

The direct antioxidant effect is based on the presence of a thiol (-SH) group in the NAC molecule. NAC is able to react with various radical and non-radical oxidants, including hydrogen peroxide, superoxide and hydroxyl anion, or peroxynitrite [34]. The indirect antioxidant effect of NAC results from the ability of NAC to be a precursor of cysteine, which is an essential glutamate and glycine for GSH formation. GSH as a direct antioxidant regulates the oxidation state in cells and at the same time acts as a substrate for several antioxidant enzymes [35]. The elucidation of the role of oxidative stress in the pathogenesis of many diseases has led to experimental or clinical use of NAC in several respiratory diseases, including chronic obstructive pulmonary disease [36], idiopathic pulmonary fibrosis [37], or pulmonary silicosis [38], as well as in variety of cardiovascular diseases [39,40], chronic nephropathy [41], or infertility [42]. In this model, intratracheal LPS, increased oxidative damage of lipids and proteins in lung tissue in accordance to other studies. Lipoperoxidation (LPO) of membrane lipids was expressed by the production of and malondialdehyde (MDA). An increase in MDA clearly showed overproduction of ROS after LPS instillation. As overproduction of reactive oxygen and nitrogen species caused by LPS-instillation may oxidize proteins, we also evaluated 3-Nitrotyrosine (3NT), a marker of protein nitration [43] which was increased after LPS.

Intravenous NAC significantly reduced MDA and 3NT in the lung homogenate, similarly to other rat model [32] and lowered LPO-markers in the lungs of rats with LPS-induced lung injury [33]. The effect of low dose NAC on MDA was almost significant (NAC10 vs. LPS, *p* = 0.051) and non-significant in terms of protein damage (3NT) (NAC10 vs. LPS, *p* = 0.073). Higher NAC dose significantly reduced oxidative damage of lipids and proteins compared to LPS group, indicating stronger effect of higher dose of NAC in protection against oxidative damage (Figure 5).

Oxidative stress induces the expression of NF-κB, which in turn leads to an increase in the production of pro-inflammatory cytokines (TNFα, IL-1β, IL-6 and IL-8). In mechanically ventilated rats, administration of LPS significantly increased all pro-inflammatory parameters (Figure 6). NAC reduces ROS-induced NF-κB activation which plays a critical role in the inflammatory cascade and immune response involved in the response to oxidative stress [44]. NAC blocks the translocation and nuclear activation of the transcription factor NF-κB, responsible for the regulation of proinflammatory gene expression. NAC has been shown to suppress the release of inflammatory cytokines TNFα, IL-1β and IL-6 in lipopolysaccharide-activated macrophages [45]. In this study, inflammation has been reduced to some extent by i.v. administration of NAC already at 10 mg/kg (IL-1β, IL-2, IL-5, IL-13 and INFγ), while higher-dose of 20 mg/kg potentiated this effect by reducing IL-1α, IL-4, IL-6, IL-10, GM-CSF and TNFα in the lung tissue. NAC also inhibits p38 MAPK kinase phosphorylation by reducing the concentration of hydrogen peroxide in the cell and adjusting the redox balance [46] and affects other pro-inflammatory mechanisms, including cyclooxygenase (COX-2), MMP-3, MMP-4, or the expression of an intracellular adhesion molecule (ICAM-1). In our study NAC10 reduced LPS-induced increase in intercellular adhesion molecule-1 (ICAM1), 4-hydroxynonenal (HNE) and myeloperoxidase (MPO), with stronger effect of NAC20 seen especially for ICAM1 (Figure 7). This effect of NAC can be assigned to the stimulation of erythroid-2-like nuclear factor (Nrf2). Nrf2 is a transcription factor that regulates antioxidant cytoprotective enzymes through a promoter sequence known as the antioxidant response element (ARE) [47].

In addition to reducing NF-κB in the lung tissue, NAC also inhibits proteolytic enzymes [48]. As proteases play a role in the LPS-induced lung damage [49], some of the protective effects given by NAC may be attributed to their inhibition. Lung oedema represented as wet/dry ratio of lung tissue was formed after LPS and significantly mitigated by NAC (Figure 4) probably due to reduced generation of ROS and other pro-inflammatory substances following NAC treatment. However, the reduction in pulmonary oedema can also be attributed to other effects of NAC, in particular on the ability to reduce pulmonary vasoconstriction and right-to-left pulmonary shunts [32,50]. NAC reduced pulmonary vascular resistance and lung oedema in dogs with oxygen-induced lung injury [50], alleviated alveolar-capillary membrane and oedema formation in contused lung [32]. The results of animal study cannot be directly applied to humans, which is a limitation of the study.

## 5. Conclusions

LPS-instilled and mechanically ventilated rats may serve as a suitable model of ARDS to test the acute effects of therapies at organ, systemic, cellular and molecular levels. Intravenous NAC, in rather low dose, improves lung functions, reduces PMNs migration into the lung, and reduces lung oedema, oxidative stress and wide range of pro-inflammatory mediators. Higher dose of NAC is more powerful as for reduction of oxidative damage and inflammation almost to baseline. The results together with literary data support the potential of NAC in ARDS.

## Data Availability

Data is contained within the article or supplementary material.

## References

[1] Mikolka P., Kosutova P., Kolomaznik M., Topercerova J., Kopincova J., Calkovska A., Mokra D. (2019). Effect of different dosages of dexamethasone therapy on lung function and inflammation in an early phase of acute respiratory distress syndrome model. Physiol. Res..

[2] Matthay M.A., Zemans R.L., Zimmerman G.A., Arabi Y.M., Beitler J.R., Mercat A., Herridge M., Randolph A.G., Calfee C.S. (2019). Acute respiratory distress syndrome. Nat. Rev. Dis. Primers.

[3] Villar J., Sulemanji D., Kacmarek R.M. (2014). The acute respiratory distress syndrome: Incidence and mortality, has it changed?. Curr. Opin. Crit. Care.

[4] Bellani G., Laffey J.G., Pham T., Fan E., Brochard L., Esteban A., Gattinoni L., van Haren F., Larsson A., McAuley D.F. (2016). Epidemiology, patterns of care, and mortality for patients with acute respiratory distress syndrome in intensive care units in 50 countries. JAMA.

[5] Villar J., Blanco J., Kacmarek R.M. (2016). Current incidence and outcome of the acute respiratory distress syndrome. Curr. Opin. Crit. Care.

[6] Chen H., Bai C., Wang X. (2010). The value of the lipopolysaccharide-induced acute lung injury model in respiratory medicine. Expert Rev. Respir. Med..

[7] Jung Y.J., Park Y.Y., Huh J.W., Hong S.B. (2019). The effect of human adipose-derived stem cells on lipopolysaccharide-induced acute respiratory distress syndrome in mice. Ann. Transl. Med..

[8] Liang Q., Lin Q., Li Y., Luo W., Huang X., Jiang Y., Qin C., Nong J., Chen X., Sooranna S.R. (2020). Effect of SIS3 on extracellular matrix remodeling and repair in a lipopolysaccharide-induced ARDS rat model. J. Immunol. Res..

[9] Bertani B., Ruiz N. (2018). Function and biogenesis of lipopolysaccharides. EcoSal Plus.

[10] Lee S.A., Lee S.H., Kim J.Y., Lee W.S. (2019). Effects of glycyrrhizin on lipopolysaccharide-induced acute lung injury in a mouse model. J. Thorac. Dis..

[11] Pooladanda V., Thatikonda S., Muvvala S.P., Devabattula G., Godugu C. (2021). BRD4 targeting nanotherapy prevents lipopolysaccharide induced acute respiratory distress syndrome. Int. J. Pharm..

[12] Yadav H., Thompson B.T., Gajic O. (2017). Fifty years of research in ARDS. Is acute respiratory distress syndrome a preventable disease?. Am. J. Respir. Crit. Care Med..

[13] Cepkova M., Matthay M.A. (2006). Pharmacotherapy of acute lung injury and the acute respiratory distress syndrome. J. Intensive Care Med..

[14] Meduri G.U., Headley A.S., Golden E., Carson S.J., Umberger R.A., Kelso T., Tolley E.A. (1998). Effect of prolonged methylprednisolone therapy in unresolving acute respiratory distress syndrome: A randomized controlled trial. JAMA.

[15] Qamar W., Khan A.Q., Khan R., Lateef A., Tahir M., Rehman M.U., Ali F., Sultana S. (2012). Benzo(a)pyrene-induced pulmonary inflammation, edema, surfactant dysfunction, and injuries in rats: Alleviation by farnesol. Exp. Lung Res..

[16] Qamar W., Khan R., Khan A.Q., Rehman M.U., Lateef A., Tahir M., Ali F., Sultana S. (2012). Alleviation of lung injury by glycyrrhizic acid in benzo(a)pyrene exposed rats: Probable role of soluble epoxide hydrolase and thioredoxin reductase. Toxicology.

[17] Kopincova J., Kolomaznik M., Mikolka P., Kosutova P., Topercerova J., Matasova K., Calkovska A., Mokra D. (2019). Recombinant human superoxide dismutase and *N*-acetylcysteine addition to exogenous surfactant in the treatment of meconium aspiration syndrome. Molecules.

[18] Mitsopoulos P., Omri A., Alipour M., Vermeulen N., Smith M.G., Suntres Z.E. (2008). Effectiveness of liposomal-*N*-acetylcysteine against LPS-induced lung injuries in rodents. Int. J. Pharm..

[19] Elsayed A., Elkomy A., Elkammar R., Youssef G., Abdelhiee E.Y., Abdo W., Fadl S.E., Soliman A., Aboubakr M. (2021). Synergistic protective effects of lycopene and *N*-acetylcysteine against cisplatin-induced hepatorenal toxicity in rats. Sci. Rep..

[20] De Flora S., Balansky R., La Maestra S. (2020). Rationale for the use of *N*-acetylcysteine in both prevention and adjuvant therapy of COVID-19. FASEB J..

[21] Davreux C.J., Soric I., Nathens A.B., Watson R.W., McGilvray I.D., Suntres Z.E., Shek P.N., Rotstein O.D. (1997). *N*-acetyl cysteine attenuates acute lung injury in the rat. Shock.

[22] Kolomaznik M., Kopincova J., Nova Z., Topercerova J., Zila I., Mikolka P., Kosutova P., Matasova K., Skovierova H., Grendar M. (2020). The effect of modified porcine surfactant alone or in combination with polymyxin B on lung homeostasis in LPS-challenged and mechanically ventilated adult rats. Molecules.

[23] Aul R., Armstrong J., Duvoix A., Lomas D., Hayes B., Miller B.E., Jagger C., Singh D. (2012). Inhaled LPS challenges in smokers: A study of pulmonary and systemic effects. Br. J. Clin. Pharmacol..

[24] Jansson A.H., Eriksson C., Wang X. (2004). Lung inflammatory responses and hyperinflation induced by an intratracheal exposure to lipopolysaccharide in rats. Lung.

[25] Blumenthal S., Borgeat A., Pasch T., Reyes L., Booy C., Lambert M., Schimmer R.C., Beck-Schimmer B. (2006). Ropivacaine decreases inflammation in experimental endotoxin-induced lung injury. Anesthesiology.

[26] Cazzola M., Calzetta L., Page C., Rogliani P., Matera M.G. (2019). Thiol-based drugs in pulmonary medicine: Much more than mucolytics. Trends Pharmacol. Sci..

[27] Rushworth G.F., Megson I.L. (2014). Existing and potential therapeutic uses for *N*-acetylcysteine: The need for conversion to intracellular glutathione for antioxidant benefits. Pharmacol. Ther..

[28] Boots A.W., Gerloff K., Bartholomé R., van Berlo D., Ledermann K., Haenen G.R., Bast A., van Schooten F.J., Albrecht C., Schins R.P. (2012). Neutrophils augment LPS-mediated pro-inflammatory signaling in human lung epithelial cells. Biochim. Biophys. Acta.

[29] Li Y., Huang J., Foley N.M., Xu Y., Li Y.P., Pan J., Redmond H.P., Wang J.H., Wang J. (2016). B7H3 ameliorates LPS-induced acute lung injury via attenuation of neutrophil migration and infiltration. Sci. Rep..

[30] Yeh S.T., Guo H.R., Su Y.S., Lin H.J., Hou C.C., Chen H.M., Chang M.C., Wang Y.J. (2006). Protective effects of *N*-acetylcysteine treatment post acute paraquat intoxication in rats and in human lung epithelial cells. Toxicology.

[31] Nagata K., Iwasaki Y., Yamada T., Yuba T., Kono K., Hosogi S., Ohsugi S., Kuwahara H., Marunaka Y. (2007). Overexpression of manganese superoxide dismutase by *N*-acetylcysteine in hyperoxic lung injury. Respir. Med..

[32] Türüt H., Ciralik H., Kilinc M., Ozbag D., Imrek S.S. (2009). Effects of early administration of dexamethasone, *N*-acetylcysteine and aprotinin on inflammatory and oxidant-antioxidant status after lung contusion in rats. Injury.

[33] Choi J.S., Lee H.S., Seo K.H., Na J.O., Kim Y.H., Uh S.T., Park C.S., Oh M.H., Lee S.H., Kim Y.T. (2012). The effect of post-treatment *N*-acetylcysteine in LPS-induced acute lung injury of rats. Tuberc. Respir. Dis..

[34] Samuni Y., Goldstein S., Dean O.M., Berk M. (2013). The chemistry and biological activities of *N*-acetylcysteine. Biochim. Biophys. Acta.

[35] Deponte M. (2013). Glutathione catalysis and the reaction mechanisms of glutathione-dependent enzymes. Biochim. Biophys. Acta.

[36] Matera M.G., Calzetta L., Cazzola M. (2016). Oxidation pathway and exacerbations in COPD: The role of NAC. Expert Rev. Respir. Med..

[37] Martinez F.J., de Andrade J.A., Anstrom K.J., King T.E., Raghu G., Idiopathic Pulmonary Fibrosis Clinical Research Network (2014). Randomized trial of acetylcysteine in idiopathic pulmonary fibrosis. N. Engl. J. Med..

[38] Sun J., Song P., Wang Y., Chen Y. (2019). Clinical efficacy of acetylcysteine combined with tetrandrine tablets in the treatment of silicosis and the effect on serum IL-6 and TNF-alpha. Exp. Ther. Med..

[39] Wilder T., Ryba D.M., Wieczorek D.F., Wolska B.M., Solaro R.J. (2015). *N*-acetylcysteine reverses diastolic dysfunction and hypertrophy in familial hypertrophic cardiomyopathy. Am. J. Physiol. Heart Circ. Physiol..

[40] Ali-Hasan-Al-Saegh S., Mirhosseini S.J., Tahernejad M., Mahdavi P., Shahidzadeh A., Karimi-Bondarabadi A.A., Dehghan A.M., Rahimizadeh E., Haddad F., Ghodratipour Z. (2016). Impact of antioxidant supplementations on cardio-renal protection in cardiac surgery: An updated and comprehensive meta-analysis and systematic review. Cardiovasc. Ther..

[41] Ye M., Lin W., Zheng J., Lin S. (2021). *N*-acetylcysteine for chronic kidney disease: A systematic review and meta-analysis. Am. J. Transl. Res..

[42] Sandhu J.K., Waqar A., Jain A., Joseph C., Srivastava K., Ochuba O., Alkayyali T., Ruo S.W., Poudel S. (2021). Oxidative stress in polycystic ovarian syndrome and the effect of antioxidant *N*-acetylcysteine on ovulation and pregnancy rate. Cureus.

[43] Rabbani N., Thornalley P.J. (2008). Assay of 3-nitrotyrosine in tissues and body fluids by liquid chromatography with tandem mass spectrometric detection. Methods Enzymol..

[44] Tenório M.C.D.S., Graciliano N.G., Moura F.A., Oliveira A.C.M., Goulart M.O.F. (2021). *N*-acetylcysteine (NAC): Impacts on human health. Antioxidants.

[45] Palacio J.R., Markert U.R., Martínez P. (2011). Anti-inflammatory properties of *N*-acetylcysteine on lipopolysaccharide-activated macrophages. Inflamm. Res..

[46] De Flora S., Balansky R., Maestra L.A.S. (2021). Antioxidants and COVID-19. J. Prev. Med. Hyg..

[47] Oter S., Jin S., Cucullo L., Dorman H.J. (2012). Oxidants and antioxidants: Friends or foes?. Oxid. Antioxid. Med. Sci..

[48] Radomska-Leśniewska D.M., Skopińska-Rózewska E., Jankowska-Steifer E., Sobiecka M., Sadowska A.M., Hevelke A., Malejczyk J. (2010). *N*-acetylcysteine inhibits IL-8 and MMP-9 release and ICAM-1 expression by bronchoalveolar cells from interstitial lung disease patients. Pharmacol. Rep..

[49] Liu X., Wang D., Zhang X., Lv M., Liu G., Gu C., Yang F., Wang Y. (2021). Effect and mechanism of phospholipid scramblase 4 (PLSCR4) on lipopolysaccharide (LPS)-induced injury to human pulmonary microvascular endothelial cells. Ann. Transl. Med..

[50] Wagner P.D., Mathieu-Costello O., Bebout D.E., Gray A.T., Natterson P.D., Glennow C. (1989). Protection against pulmonary O_2_ toxicity by *N*-acetylcysteine. Eur. Respir. J..

